# Supporting youth 12–24 during the COVID-19 pandemic: how Foundry is mobilizing to provide information, resources and hope across the province of British Columbia

**DOI:** 10.1177/1757975920984196

**Published:** 2021-03

**Authors:** Marco Antonio Zenone, Michelle Cianfrone, Rebecca Sharma, Sanaa Majid, Jasmine Rakhra, Kathryn Cruz, Stefanie Costales, Monique Sekhon, Steve Mathias, Andrew Tugwell, Skye Barbic

**Affiliations:** 1 BC Children’s Hospital, Vancouver, British Columbia, Canada; 2 Foundry, Vancouver, British Columbia, Canada; 3 Department of Occupational Science and Occupational Therapy, Faculty of Medicine, University of British Columbia, Vancouver, British Columbia, Canada; 4 Providence Health Care Research Institute, Vancouver, British Columbia, Canada; 5 Centre for Health Evaluation Outcome Sciences, Vancouver, British Columbia, Canada

**Keywords:** adolescents and youth, health literacy, mental health

## Abstract

Foundry is a province-wide network of integrated health and social service centres for young people aged 12–24 in British Columbia (BC), Canada. Online resources and virtual care broaden Foundry’s reach. Its online platform – foundrybc.ca – offers information and resources on topics such as mental health, sexual wellness, life skills, and other content suggested by youth and young adults. The COVID-19 pandemic has presented significant and unique challenges to the youth and their families/caregivers served by Foundry. Disruptions to school, access to essential healthcare services such as counselling, familial financial security and related consequences has left young people with heightened anxiety. The Foundry team mobilized to respond to these extenuating circumstances and support BC youth and their families/caregivers during this hard time through three goals: (1) to amplify (and translate for young people and their families/caregivers) key messages released by government to support public health responses to the COVID-19 pandemic; (2) to develop content that supports the needs of young people and their families/caregivers that existed before COVID-19 and are likely to be exacerbated as a result of this pandemic; and (3) to develop and host opportunities through social media and website articles to engage young people and their families/caregivers by creating a sense of community and promoting togetherness and social connection during the COVID-19 pandemic. Each goal and plan integrated the leadership, feedback and needs of youth and their families through engagement with Foundry’s provincial youth and family advisory committees. Our study evaluated Foundry’s media response to the COVID-19 pandemic by recording/measuring (1) the website/social content created, including emerged thematic topic areas; (2) the process of topic identification through engagement with youth and young adults; (3) the social and website analytics of the created content; and (4) the constant, critical team-reflection of our response to the pandemic. Following measurement and reflection, our team offers recommendations to health promotion organizations for future preparedness.

## Background

Foundry is a province-wide network of integrated health and social service centres for young people aged 12–24 in British Columbia (BC), Canada and is supported administratively by the Foundry central office team at Providence Health Care. Foundry Vancouver Granville opened in March 2015 and served as the prototype centre informing Foundry’s model. Since then, the Foundry network has grown to include 11 centres, with another eight expected to open by 2022, operating in both urban and rural communities. Between April 2018 and September 2020, Foundry served 18,375 unique youth and young adults in BC, providing 107,708 services over 82,324 visits. Online resources and virtual care further broaden Foundry’s reach. Its online platform – foundrybc.ca – offers information and resources on mental health, sexual wellness, life skills, and other content suggested by youth and young adults. Foundry Virtual offers young people and their caregivers across BC drop-in counselling, peer support and primary care through voice, video and chat. Virtual services were in development prior to the pandemic and became available in April 2020.

The COVID-19 pandemic has presented significant and unique challenges to the youth and family/caregivers served by Foundry. In BC, there have been 9956 COVID-19 cases, 819 hospitalizations, and 244 deaths as of 7 October 2020 (1). Among youth and young adults aged 10–19, 585 cases have been reported, increasing to 2278 cases for those aged 20–29 (1). Mitigation measures to reduce the spread of COVID-19, such as physical distancing and the subsequent closure of businesses, led to the loss of 400,000 jobs in March and April 2020 alone (2–4). On 17 March 2020, Premier John Horgan declared public schools were indefinitely suspending in-class instruction (5). In early June, schools gradually offered in-class instruction on a voluntary, non-mandatory basis (6). The usual mental health or medical services youth and their families access – such as counselling – paused for an indefinite period until available through digital delivery systems. Recreational opportunities, namely recreational centres and their programming, sports leagues, arts-based classes, and related opportunities, were closed (7,8). Other important life events such as graduations and driver licensing either did not happen this year, took a different format, or have been delayed (9–11). These disruptions have left youth and young adults, along with their families/caregivers, with heightened stress, anxiety and uncertainty.

The mental health impacts of the pandemic are becoming increasingly recognized, especially among the younger demographic (12–18). While specific figures reporting the mental health impacts of the pandemic are not available for younger BC audiences, a Canada-wide survey suggests that younger Canadians are reporting higher levels of anxiety (19). A national survey conducted in early May 2020 suggests that 31.2% of Canadians aged 18–39 experienced moderate to severe anxiety (20). Approximately 72% of youth worried about someone in their immediate family catching the virus, while 49% worried about catching the virus themselves (21). Among youth fearful of catching the virus, higher levels of anxiety and sleep disturbances were reported (21). In other countries, such as the United Kingdom, a survey of youth and young adults found that 83% felt the pandemic had worsened their condition, and 26% could not access mental health support (22). Further, during economic downturns, as described by Golberstein and colleagues, the mental health of youth and young adults is likely to worsen due to the financial struggle of caregivers and result in possible cascading effects (23). Other studies describe that during an emergency, people who were previously living with a mental health condition may be more susceptible to either relapsing or having their mental health further deteriorate (24). Increased mental health risks during the COVID-19 pandemic have led to a global research call for rapid responses to consider the long-term mental health needs of young people (25).

Existing investments and action to support the mental health of youth and adults by the Canadian and BC governments, while laudable, are primarily for service-oriented initiatives. For example, the Prime Minister of Canada, Justin Trudeau, announced a CAD$7.5 million investment to Kids Help Phone to provide additional funding for crisis management and mental health support (26) while BC’s Minister of Mental Health and Addictions, Judy Darcy, announced a CAD$5 million investment to expand mental health support across BC in response to the pandemic (27). Other services promoted include various helplines, chat services, resource hubs, or COVID-19 spread mitigation information (28). Although we recognize that crisis services are needed, little consideration was given to how to provide wrap-around services to support and coordinate the short-, intermediate-, and long-term needs of young people and their families.

With a province-wide network of health and social service centres and virtual service offerings that operate on a common stepped-care model, Foundry was conceived to provide a range of integrated services to support the diverse needs of young British Columbians and their families/caregivers. From upstream health promotion, to early and tertiary interventions, Foundry’s model has allowed for a unique response to the pandemic.

While prioritizing the delivery of services through its virtual platform, Foundry crafted and reframed our role during the pandemic as *supporting youth through it, versus becoming COVID-19 experts.* In the context of stressful news headlines, disruptions to lives, and uncertainty, Foundry transformed its social media and website content into a trusted platform to support youth and young adults and their families/caregivers to preserve their mental health through social connection, engagement, and advice to make sense of the state of the world. With content, led most often by youth, Foundry provided and continues to provide opportunities for trusted information, resources and meaningful social connection. In addition, Foundry translated new information to our audience in an accessible format.

In this paper, we report on the development of Foundry’s pandemic response as a unique, health-promoting, provincial organization, evaluating our actions and providing recommendations for other organizations based on our learning and experience.

## Methods

### Foundry COVID-19 organizational response

In the lead up to the World Health Organization pandemic declaration on 11 March 2020, Foundry created the ‘COVID-19 Communications Response Team’ that included representatives from its communications, clinical, knowledge exchange, and youth and family engagement teams, as well as health literacy representatives from BC Children’s Hospital. Created on 28 February 2020, the team rapidly distilled information from the provincial government’s public health authority, news media, non-governmental organizations, and non-profits, as well as new information from its network of lead agencies, youth and family advisors. Foundry’s role on how best to support youth, young adults and families/caregivers emerged. After an initial series of meetings in early March 2020, the team crafted three goals.

The first goal was *amplification and translation of crucial government messages*, aimed at supporting the dissemination of critical measures to reduce the spread of COVID-19. The second goal was the *development of content to support the needs of young people and caregivers that existed before COVID-19 and were likely to be exacerbated as a result of this pandemic*. For example, a young person struggling with anxiety may experience heightened anxiety due to the stress and have limited opportunities to connect with counsellors or other supports. The third goal was the *development of hosting opportunities, through social media and website articles, to engage young people and caregivers, creating a sense of community by promoting togetherness and social connection during the COVID-19 pandemic*. The intention of the last goal was to support our audiences’ mental health through health promotion interventions. In its provincial leadership role, Foundry was well situated to support young people and their families during the COVID-19 pandemic by actioning our understanding of their context and providing practical support.

Following the establishment of these goals, the team developed a public website page on 17 March 2020 on Foundrybc.ca designed to provide up-to-date and accessible information on COVID-19 to Foundry’s primary audiences, young people aged 12–24 and their families/caregivers (29). The communications response team then met daily to discuss opportunities for engagement and social connection for young people and their families/caregivers in a time of physical distancing. Foundry also formed a separate social media working group on 7 April 2020, who met twice a week to plan and develop content that would meet the goals. The team generated an initial list of potential content and topics that was then brought to Foundry’s provincial youth and family advisory groups for feedback.

### Engaging young people, caregivers and partners: content development

To develop topical content reflecting the priorities of Foundry’s audiences, engagement of youth, young adults, and their families was done through several Foundry engagement groups. During the pandemic, advisory groups composed of young people and family members/caregivers identified topics for the communications team to pursue and reviewed content ideas generated by the communications response team and social media working group for appropriateness and suggested improvements. For example, Foundry liaised with a young person to improve an article by identifying activities to engage in while self-isolating. Content pieces written by a communications team member or someone who was not a youth or young adult were reviewed by a young person before publication on the website or social media platform. In several cases, the advisory group members led the response by drafting the content on their own, which was later reviewed by a communications team member. Foundry’s Youth Peer Engagement Coordinator and Family Engagement Coordinator facilitated weekly discussions with youth and family/caregiver advisors to respond to current events and led engagement work with these groups.

The team also liaised with community partners from across BC in content planning. The communications response team received content suggestions from Foundry’s network of lead agencies from 11 diverse communities, government agencies and other non-profits and mental health organizations. The team established evaluation criteria to select which content to share or translate for a youth or family/caregiver audience to ensure the content was youth-focused, evidence-informed and relevant to the BC context.

### Response evaluation

Buffer, a social media analytics platform, and Google Analytics were used to report on the reach of our COVID-19 associated content. Metrics were tabulated from 4 March 2020 to 1 June 2020. The rationale for date selection was that in early March, Foundry began preparation for COVID-19 response planning. The end date of 1 June 2020 represents the first phase ‘reopening’ date announced by the British Columbian Government (30). Metrics were reported from Foundry’s website and three social media sites: Facebook, Instagram, and Twitter. The metrics included daily average engagements, daily average impressions, daily average clicks, total number of post impressions, post reach, total number of link clicks, total number of reactions, total number of shares, total number of likes, engagement rate, top gender and age for people reached, Instagram stories total, Instagram stories impressions, Instagram stories reach, Instagram stories engagement rate, number of retweets, and number of engagements.

In order to evaluate Foundry’s processes, procedures, and organizational features that supported or acted as barriers to the pandemic response, the team took part in many internal discussions around common themes. Identified successes, as well as areas of improvement are detailed in the following section.

## Results

### Content created

From the pandemic declaration until 1 June 2020, five website content pieces and a cumulative 132 social media posts across all platforms were developed to support youth and young adults aged 12–24 and their caregivers. Website content created included an overview of COVID-19 in the form of a youth-friendly overview page, information about sex during COVID-19, a guide for parents and caregivers to talk about COVID-19 to their youth or young adults, tips for when ‘normal’ does not feel normal, and information about physical distancing and its importance. All content was sent to a plain language specialist as needed, to ensure accessibility for Foundry’s target audiences. Social media posts explored themes directing youth to website content, infographics about COVID-19, resources about health service access (such as information regarding Foundry Virtual), tips for preserving social connection during the pandemic, and reassuring messages of support.

### Social media and website analytics

COVID-19 content on foundrybc.ca received high web traffic. The highest performing article, ‘Physical distancing and why it helps during a pandemic’, received 12,225 page views from 11,145 sessions and 10,111 unique users. The foundrybc.ca COVID-19 home page, ‘COVID-19 (novel coronavirus) information’ received 1847 page views from 829 sessions and 1299 unique users. The ‘Sex and COVID-19’ information page received 265 page views from 128 sessions and 209 unique users, and the article for caregivers, ‘How to talk to your teens about the coronavirus’ received 224 page views from 82 sessions and 176 unique users. Last, the ‘Tips for when “normal” does not feel normal anymore’ article received 77 page views from 60 sessions and 58 unique users. Other Foundrybc.ca webpages that were featured but not created specifically for COVID-19, received a total cumulative viewership of 6822. On average, users spent 2.18 minutes on Foundrybc.ca and viewed 2.18 pages. Approximately 66.6% of users were female, while 33.4% were male (Google Analytics could only record 32.09% of user gender due to data collection restrictions). Users were primarily from Canada (65.5%), followed by the United States (20%).

On Facebook, 56 posts were shared, which resulted in 617,600 post impressions and reached 460,400 persons. Page and post engagements totalled 17,200 and 5538 link clicks. There were 2096 reactions and 707 shares, giving an engagement rate of 5.54%. The average daily engagement per post was 191 engagements, while there were 6868 daily average impressions. On average, posts directed 62 clicks to foundrybc.ca. Posts receiving high views on Facebook included the announcement of Foundry Virtual (36,935 impressions), key COVID-19 phone numbers (3058 impressions), and information on virtual drop-in counselling (2918 impressions). On Instagram, 42 posts resulted in 184,100 impressions, reaching approximately 116,900 people and 1746 likes. The daily average impressions totalled 2045, and each post averaged 42 likes. The engagement rate of content was 5.79%. On Instagram stories, 139 stories resulted in 33,300 impressions and a reach of approximately 28,000 persons, with a low engagement rate of 0.06%. Popular posts on Instagram included a post on sex and COVID-19 (1307 impressions), virtual launch information (1197 impressions) and physical distancing information (1059 impressions). There were 34 posts on Twitter, resulting in 33,100 impressions, 171 retweets, 1078 engagements, 128 clicks and 225 likes. The average impression per tweet was 974 impressions; the average engagement per tweet was 32 engagements, delivering an engagement rate of 2.88%. Top performing posts on Twitter included information about Foundry Virtual services (3329 impressions) and self-care bingo (3009 impressions).

### Team reflection: successes

The Foundry response team identified several facilitating factors that allowed an effective response to the pandemic. First, the organizational structure of Foundry, including its partnership with the BC Children’s Hospital Health Literacy Team, allowed for rapid goal and content development. Immediately, the team mobilized to create a COVID-19 Communications Response Team that met regularly. Previous Foundry communication strategies, social media policies and procedures, and links to youth, advisory groups, and clinical expertise were embedded within the organizational structure. This allowed for the intentional creation and integration of teams in response to emergencies within our larger communications plan and for streamlined decision-making processes. The adaptability of the team and internal structure allowed for the re-prioritization of different work areas for a more fulsome, intentional and efficient response.

Second, the pandemic response goals, strategy and actions were informed and reliant on youth, young adults and families/caregivers. As an organization that exists to support these groups, their perspective, feedback, contributions and leadership were necessities for guiding the response. The internal structure, and prioritization of meaningful youth and caregiver engagement, allowed pandemic-related content to be primarily designed or reviewed by the audience for which it was designed. The success of several content areas – namely the physical distancing article (https://foundrybc.ca/stories/social-distancing-and-why-its-the-best-thing-to-help-a-pandemic/) – is an example of how engagement successfully delivered content on relevant and essential topics for youth and young adults. A structured and supportive environment for the youth and family/caregiver advisors allowed for content suggestions, ideas and development. Staff found ways to ensure young people’s and families’/caregivers’ perspectives were safely and meaningfully heard.

Third, Foundry’s response was informed by and in partnership with organizations from across BC, such as the Ministry of Health, Provincial Health Services Authority, and the BC Centre for Disease Control. As Foundry centres rely on partnerships with local communities across the province, diverse groups of persons with different geographic contexts also contributed to the content ideas. The COVID-19 content was regularly promoted by other health services and youth health organizations, as well as by prominent politicians, government officials, epidemiologic organizations, and staff from school districts. While these other partners promoted Foundry content, the team also shared content from other organizations, such as content from Health Canada. This allowed for widespread dissemination of resources.

### Team reflection: areas of improvement

After evaluation of the response thus far, the Foundry team has taken several learnings forward to improve our pandemic efforts and for future emergency responses. First, while engagement was essential, it also revealed several limitations of existing youth and family engagement processes. The youth engaged were facing the same difficulties as the general audience Foundry serves (i.e. stress around work/study). In an uncertain environment, there were occasions of confusion for both the team and among young people regarding the engagement process, which suggests the need for more defined processes throughout engagement. In addition, the period in which content needed development overlapped with the final exam period of many of the youth and young adults. While such considerations would not be an issue under normal circumstances, in the pandemic context, this had an impact on their capacity and availability to develop and review content. This is an opportunity for Foundry to reflect, identify supporting infrastructure and clarify internal policies and processes around engagement. This will support the transparency and clear communication of reciprocal expectations for youth and family advisors and Foundry, and the development of a more efficient strategy for our pandemic response and future emergency preparedness. Families/caregivers similarly experienced a higher burden of regular life duties during this time (i.e. homeschooling, working from home), which had an impact on their capacity and ability to engage with Foundry. Again, this is a lesson learned: to ensure that there are engagement plans and processes in place that take into account emergent context shifts.

Second, there were challenges publishing content in a timely way. The process of creating content with accurate information and ensuring the delivery of such content was accessible through youth and family review was time-consuming. Often, government advice changed during the process of such reviews. For example, while creating resources on social distancing, the recommendations from the BC government changed with the development of the pandemic. Finally, the internal team faced difficulties adapting to the COVID-19 pandemic context. Like other organizations, the team transitioned from in-person meetings to meeting virtually over a short period. The uncertain environment posed by COVID-19 made work-planning difficult amid ever-changing workloads. The lessons learned from this experience will be helpful in future situations where remote work is warranted.

## Discussion

This analysis of Foundry’s response to the COVID-19 pandemic adds to the literature on using websites and social media to support particular groups to get through times of emergency. The actions taken by Foundry supporting connection to resources, the creation of information, and opportunities to increase engagement supported youth, young adults, and their families during this unprecedented time. Other studies such as Calder *et al*. and Hoffer *et al*. found similar conclusions in that social media offers a useful tool to support an emergency response (31,32). The information provided on such platforms is valuable evidence-based information delivered in a friendly, accessible format (33). In the pandemic context, and applicable to other issues, this is important to reduce the spread of misinformation (34). The changing role of social media in health promotion and its increasing usability is being recognized and applied, especially when delivering health promotion messages to large geographic areas when in-person initiatives are not feasible or possible (35,36). As health promoters continue to utilize social media for its advantageous qualities, such as the opportunity for widespread message dissemination in a short time period, more scholarly attention is needed to examine the drawbacks of such approaches.

As a leading health-promoting organization, several recommendations are offered for future health promotion, emergency-context, preparation planning. First, in emergency contexts, it is recommended that organizations define the roles and responsibilities of the response team, as well as those of the youth and family/caregiver advisors. Uncertainty regarding task assignments may lead to delayed action, therefore, a designated project manager assigned to oversee the response is required. Second, development of a clear and concise communications strategy is recommended. This would allow for comprehensive communication with the audiences the organization serves, as well as help the team plan the response in a strategic, coordinated and proactive way. Third, investing in well-funded engagement teams and youth- and family-centred platforms/technologies is recommended. Although the Foundry response was primarily led and informed by youth, young adults and families/caregivers, proactive planning and resources may have enhanced our overall experience and impact. We recognize the critical importance of these stakeholder groups leading this work and the need to provide training and support to develop their expertise. Moving forward, provision of honorariums and creation of opportunities for ongoing engage-ment, versus one-off, reactive engagements are recommended. It is advantageous to allow the target audience to take leadership roles in amplifying content and sharing learning. The selection of project management software and communication tools should be undertaken early in the project after communal discussion with engagement groups; the necessary training should subsequently be provided.

Fourth, establishment of clear criteria to support selecting content to either translate or amplify from partner agencies is recommended. Establishing criteria for sharing content either on social media or a website is needed to ensure information is accurate and that the organization creating such information is credible. Fifth, organizations should proactively hire young people and provide opportunities for them to write about their own experience or topic-specific information articles. In this arrangement, as described by King *et al*., young people could contribute their perspective to articles while receiving the support of senior staff (37). Thus, the organization would be equipped to prepare content in any scenario. Sixth, it is imperative to evaluate the organizational response. Evaluation should occur both during and following an emergency. Such information is key to ensuring the response is in fact addressing the goals of the organization’s strategy. Evaluation is also useful for future situations, and dissemination of the findings, either through traditional academic formats and/or to partners, the audience served, and the public.

In evaluating Foundry’s response, there were several limitations. First, due to restrictions on data collection among minors, available metrics could not report the specific demographics of visitors to foundrybc.ca who were under 18 years of age. Therefore, while the total number of visitors to specific webpages is reported, it is not possible to state with certainty the age of those accessing such pages. The social media analytics, similar to the website content, could not confirm the demographic details of all audiences who viewed or engaged with the social content. Second, the results presented are confined to website and social media metrics. Thus, qualitative input is needed to confirm how such messages were received. Finally, the cumulative social media metrics, such as impressions, include all views to the posts during the data collection period (unless specified for a particular post). While most posts shared were specific to the pandemic, several were not directly related.

## Conclusion

The experience of Foundry in responding to the COVID-19 pandemic is an important health promotion example that can serve other organizations in emergency planning. This paper describes the process of an interdisciplinary communications team responding to and establishing organizational goals to support youth, young adults and families/caregivers in times of emergency. Based on the Foundry team experience, several recommendations are offered for other organizations to consider for emergency preparation. Organizations serving youth and young adults should prioritize youth engagement, measurable goals for emergency responses, and plans to mobilize an emergency response team in times of a pandemic.
